# FAM3A Ameliorates Brain Impairment Induced by Hypoxia–Ischemia in Neonatal Rat

**DOI:** 10.1007/s10571-021-01172-6

**Published:** 2021-12-01

**Authors:** Qing Song, Qingying Gao, Taotao Chen, Ting Wen, Peng Wu, Xiao Luo, Qiao Yi Chen

**Affiliations:** 1 Department of Obstetrics and Gynecology, The First Affiliated Hospital of Xi’an Jiaotong University, Xi’an, 710049 Shaanxi China; 2 Department of Cell Biology and Genetics, School of Basic Medical Sciences, Xi’an Jiaotong University, Xi’an, 710049 Shaanxi China; 3 The Third Affiliated Hospital of Xi’an Medical University, Xi’an, 710049 Shaanxi China; 4 Department of Physiology and Pathophysiology, Xi’an Jiaotong University Health Science Center, Xi’an, 710061 Shaanxi China; 5 Institute of Neuroscience, Translational Medicine Institute, Xi’an Jiaotong University Health Science Center, Xi’an, 710061 Shaanxi China

**Keywords:** Brain, Hypoxia–ischemia, FAM3A, TNF-α, IFN-γ, Mitochondria

## Abstract

**Abstract:**

Hypoxia–ischemia (HI) during crucial periods of brain formation can lead to changes in brain morphology, propagation of neuronal stimuli, and permanent neurodevelopmental impairment, which can have profound effects on cognitive function later in life. FAM3A, a subgroup of family with sequence similarity 3 (FAM3) gene family, is ubiquitously expressed in almost all cells. Overexpression of FAM3A has been evidenced to reduce hyperglycemia via the PI3K/Akt signaling pathway and protect mitochondrial function in neuronal HT22 cells. This study aims to evaluate the protective role of FAM3A in HI-induced brain impairment. Experimentally, maternal rats underwent uterine artery bilateral ligation to induce neonatal HI on day 14 of gestation. At 6 weeks of age, cognitive development assessments including NSS, wire grip, and water maze were carried out. The animals were then sacrificed to assess cerebral mitochondrial function as well as levels of FAM3A, TNF-α and IFN-γ. Results suggest that HI significantly reduced FAM3A expression in rat brain tissues, and that overexpression of FAM3A through lentiviral transduction effectively improved cognitive and motor functions in HI rats as reflected by improved NSS evaluation, cerebral water content, limb strength, as well as spatial learning and memory. At the molecular level, overexpression of FAM3A was able to promote ATP production, balance mitochondrial membrane potential, and reduce levels of pro-inflammatory cytokines TNF-α and IFN-γ. We conclude that FAM3A overexpression may have a protective effect on neuron morphology, cerebral mitochondrial as well as cognitive function.

**Graphical Abstract:**

**Figure:**
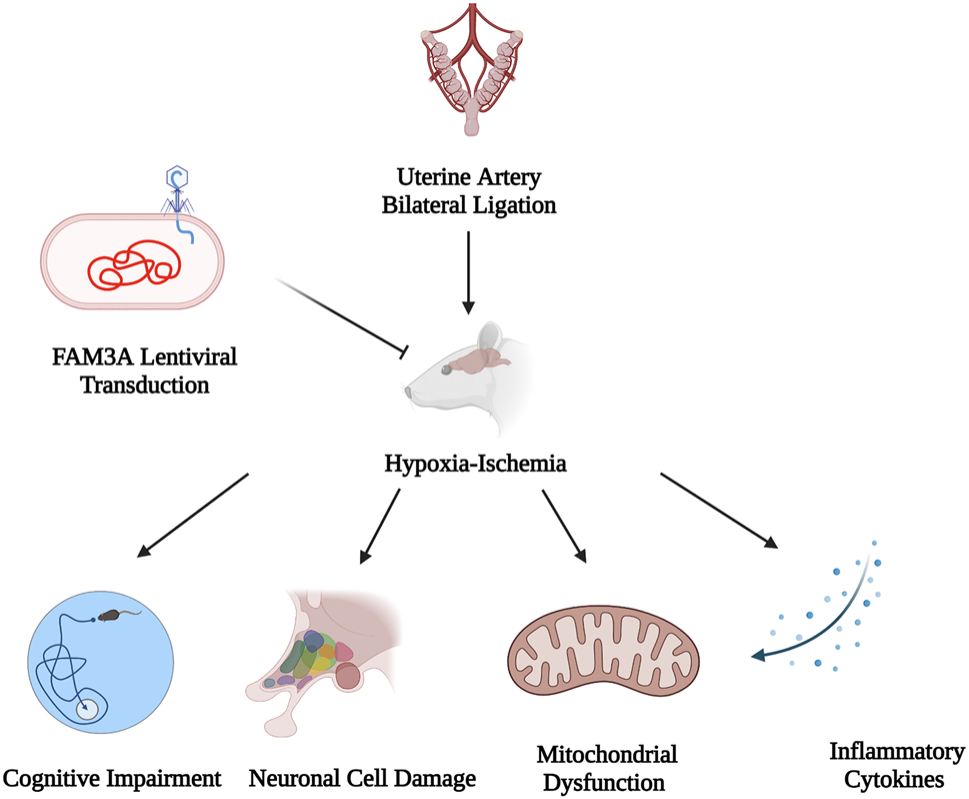
Created with Biorender.com

## Introduction

Neonatal hypoxia–ischemia (HI) is clinically defined as “asphyxia of the umbilical blood supply to the human fetus occurring at 36 gestational weeks or later” (Millar et al. 2017; Shah et al. 2006; Volpe 2012). Ischemia occurs as a result of blood obstruction, which can lead to hypoxia (reduced oxygen) in tissues or the body. Brain HI is one of the most prevalent complications as a result of obstructed blood flow and impaired brain metabolism. Arrest of intrauterine blood flow as in the case of HI, can lead to irreversible physiological changes such as neuronal destruction, which can lead to permanent cognitive dysfunctions including deteriorated learning and memory (Nalivaeva et al. 2018; Babenko et al. 2015; Desplats 2015). Current evidence suggests that a myriad of factors such as smoking, drug administration, alcohol consumption as well as diet and infectious diseases may have crucial roles in inducing gestational HI (Nalivaeva et al. 2018; Faa et al. 2014; Charil et al. 2010; Li et al. 2012; Monk et al. 2013; Donald et al. 2015; Gawalek and Sliwowska 2015; Kohlmeier 2015; Labouesse et al. 2015). Fetal distress as a result of neonatal HI accounts for 23% of infant mortality worldwide, affecting up to 1.2 million infants annually (Lawn et al. 2005). While advanced intensive care has improved overall infant mortality rate, limited therapeutic strategies licensed for neonates hamper effective intervention, leading to a substantial rise in morbidity and disease burden in the adult population. In surviving infants, disability rate remains high throughout life. HI-induced brain injury can lead to long-term sensory, motor, and cognitive impairment such as cerebral palsy, mental retardation, seizures, hearing and vision loss, microcephaly, muscle spasticity, and language disorders, which immensely affect quality of life (Millar et al. 2017; Robertson and Finer 1985; Shankaran et al. 1991, 2012). In adult mouse model, Feng et al. demonstrated that HI can result in significant neuronal loss and cognitive deficits (Feng et al. 2020). A recent meta-analysis study showed that hypothermia may only be effective in reducing death and neurological handicaps at 18 months follow-up, but not enough for preventing all neurological injuries (Tagin et al. 2012). Global prevalence of neonatal HI-induced brain injuries and related poor long-term outcomes underscore the pressing need for molecular mechanistic research and novel neuroprotective measures.

Both clinical and molecular studies have suggested that loss of mitochondrial function, which plays a central role in cerebral energy metabolism, is a determining factor in neuronal cell survival. And notably, mitochondria function varies differently in the developing brain compared to the matured brain (Robertson et al. 2006), which prompted our interest to examine mitochondria dysfunction in HI-related brain injury. Under HI condition, even short-term blockage of blood supply can impair mitochondrial function and oxidative phosphorylation, which can rapidly induce neuronal cell death, and irreversibly disrupt neuronal dendrites and synaptic structures (Feng et al. 2020; Ten et al. 2004). Current evidence suggests that FAM3A, a member of the family with sequence similarity 3 (FAM3) gene family, may play a role in neuronal cell apoptosis and mitochondrial dysfunction, both of which are critical for normal brain development (Liu et al. 2019; Song et al. 2016). FAM3 is a family of cytokine-like genes with four identified members: FAM3A, FAM3B, FAM3C, and FAM3D (Xu et al. 2019; Yang and Guan 2013; Zhu et al. 2002). FAM3A protein is composed of 230 amino acid residues and is found in most tissues (Song et al. 2015; Zhu et al. 2002). As a target of peroxisome proliferator-activated receptor γ (PPAR γ), FAM3A has been shown to inhibit hyperglycemia and insulin resistance via the PI3K/AKT signaling pathway (Song et al. 2017; Wang et al. 2014). Biologically, FAM3A can localize in the mitochondria, and is known for promoting adenosine 5′-triphosphate (ATP) production and mitochondrial respiration in neuronal cells (Song et al. 2015, 2016; Jia et al. 2014). Under hypoxic conditions, overexpression of FAM3A positively regulates angiogenesis via activation of VEGFA and promotes endothelial tube formation (Xu et al. 2019). In addition, FAM3A has been shown to protect chondrocytes from iterleukin-1B-induced apoptosis (Yan et al. 2019). Previous studies in our lab have reported the role of FAM3A in protecting neuronal hippocampal HT22 cells against oxidative stress-induced, as well as mitochondrial dysfunction-elicited apoptosis via the PI3K/AKT and CHOP-Wnt pathways, respectively (Song et al. 2015, 2016). Moreover, we also showed that FAM3A can alleviate glutamate-induced toxicity in pheochromocytoma PC12 cells by preserving calcium homeostasis (Song et al. 2017). In this current study, we aim to explore the protective roles of FAM3A in HI-induced brain impairment, especially through regulation of mitochondrial function. Specifically, we performed uterine artery bilateral ligation on pregnant rats to induce HI in the neonates, followed by overexpression of FAM3A via postnatal lentiviral injection. Results in this study indicate that HI significantly reduces FAM3A expression in rat brain tissues, and that overexpression of FAM3A effectively improved cognitive and motor functions in HI rats as reflected by improved NSS evaluation, cerebral water content, limb strength, as well as spatial learning and memory. At the molecular level, FAM3A was able to promote ATP production, balance mitochondrial membrane potential, and reduce levels of pro-inflammatory cytokines tumor necrosis factor (TNF-α) and interferon gamma (IFN-γ).

## Materials and Methods

### Animals

Time-mated pregnant Sprague Dawley (SD) rats were purchased from Beijing Weitong Lihua laboratories (*n* = 12). After being transferred to Xi’an Jiaotong University, the rats were group-housed prior to surgery and maintained at 55% humidity, 22 °C, and in a 12 h light/dark cycles. The animals were allowed free access to water and rat chow. All experimental procedures on animals were approved by and conducted in accordance with Xi’an Jiaotong University’s regulations and legal requirements.

### Uterine Artery Bilateral Ligation

On day 14 of gestation (22 days at term), the maternal rats were anesthetized using ketamine (100 mg/kg) and midazolam (5 mg/kg). After midline laparotomy, the uterine artery was exposed and both uterine arteries were ligated. Next, the uterus was placed back into the abdominal cavity, and closed using Vicryl suture. The animals recovered within 4–8 h and received free access to water and rat chow. All pregnant rats delivered spontaneously, and the litters were randomly culled to 6 immediately after birth to ensure equal number of fetuses between the three experimental groups (*n* = 6 for each group) [non-HI with control lentivirus (Ctrl-LV-Ctrl), HI with control lentivirus (HI-LV-Ctrl), and HI with FAM3A lentivirus (HI-LV-FAM3A)]. The newborn pups were then transferred and remained with untreated foster mothers until weaning. At 6 weeks of age (sexually mature) (Andreollo et al. 2012), the animals went through a series of behavioral tests and were then sacrificed under anesthesia and brain tissues were extracted for the remaining experiments.

### Culture and Transduction of HEK-293T Cells

HEK-293T cells purchased from the Institute of Biochemistry and Cell Biology were cultured in DMEM supplemented with 10% FBS, 100 µg/ml streptomycin, and 100 U/ml penicillin at 37 °C and 5% CO_2_. The coding sequence of FAM3A was amplified by RT-PCR: forward 5′-TCATGAGCAGCGTCAAAGAC-3′ and reverse 5′-AGGGTACCTTCATGCAGTGG-3′. PCR fragments and the pGC-FU plasmid were Age-I-digested and ligated with T4 DNA ligase, and cloned into pGC-FU-FAM3A. After reaching80% confluency in 10 cm dishes, HEK-293T cells were co-transfected with a pGC-FU plasmid (20 µg) with cDNA encoding FAM3A, pHelper1.0 plasmid (15 µg) and pHelper 2.0 plasmid (10 µg) using Lipofectamine 2000 (100 µl) to generate Lentivirus LV-FAM3A cells. A control lentiviral vector expressing GFP alone (LV-Ctrl) was also generated. The viral stocks were diluted to 1 × 10^8^ TU/ml and stored at  − 80 °C.

### In Vivo Lentiviral Vector Gene Transfer

Prior to injection, viral stocks were thawed on ice and resuspended by pipetting. The animals were anesthetized using ketamine (100 mg/kg) and midazolam (5 mg/kg). LV-FAM3A vectors were stereotaxically injected into the striatum of 1-week-old HI pups (*n* = 6) at + 0.5 mm and − 0.25 mm rostral to bregma in both hemispheres. Non-HI (*n* = 6) as well as the other HI group (*n* = 6) of pups were administered with LV-Ctrl vectors. The injections were administered with a 34-gauge blunt-tip needle using an automatic injector at 0.2 µl/min. After the injection, the skin was sutured with 6–0 Prolene sutures.

### Neurological Severity Score

The neurological severity score was based on a 10-point system (Ando et al. 2011). Higher scores reflect more severe neurological impairment. A battery of 10 tasks (0 for success, 1 for failure) were included: exit circle, mono-hemiparesis, straight walk, startle reflex, seeking behavior, beam balancing, round stick balancing, beam walk (3 cm), beam walk (2 cm), and beam walk (1 cm). Exit circle indicates ability to exit a circle of 30 cm in diameter under 3 min, mono-hemiparesis indicates paresis of upper and/or lower limb of the contralateral side, straight walk indicates ability to walk straight when animal is put on the floor, startle reflex indicates innate reflex in response to loud hand clap, seeking behavior indicates physiological “interest” in the surrounding environment, beam balancing indicates ability to balance on a 7 mm width beam for at least 10 s, round stick balancing indicates ability to balance on a 5 mm round stick for at least 10 s, and beam walk indicates ability to cross a 3 cm, 2 cm, and 1 cm wide beam. Scoring was performed when the animals reached 6 weeks of age.

### Hanging Wire Grip Test

The animals suspended their bodies with their forelimbs on a wire that is 40 cm in length, 0.3 cm in diameter, and 45 cm above ground. Performance on the grip test was based on a five-point score system where 0 = fall, 1 = one or two forelimbs holding tightly on the wire, 2 = attempt to climb on the wire, 3 = one or two forelimbs in combination with one or two hindlimbs holding tightly on the wire, 4 = holding onto the wire with forelimbs, hindlimbs, and tail, and 5 = successful escape off of the wire. Time spent holding onto the wire was also categorized based on a five-point system where 1 ≤ 10 s, 2 = 11–30 s, 3 = 31 s–2 min, 4 = 3–5 min, and 5 ≥ 5 min.

### Morris Water Maze Test

The animals were randomly placed inside the pool that is 1.2 m in diameter, 0.5 m in height, and filled up to 35 cm in depth. The water temperature was set at 22 ± 2 °C. Each animal took three trials for 5 days, each trial lasted for 60 s. The starting quadrant for each animal varied randomly. As part of the training, the animals were placed at a random start position facing the tank wall and allowed 60 s to find the hidden platform (located in a fixed position 1.5 cm below the surface of the water) to escape the water maze. Those that failed to find the platform were assigned a latency score of 60 s. All animals were left on the platform for 15 s before the start of the next trial. At the end of the trial, the animals were wiped dry and returned to their home cage. Escape latency was recorded using Animal video tracking analysis system AVTAs Version 3.0 single tracking by a blinded assistant (AniLab, Ningbo, China).

### Cerebral Water Content Measurement

The brain was quickly removed from sacrificed rats, and the ipsilateral basal ganglia sample was taken and immediately weighed on an electronic balance (model ME204, Mettler) for wet weight measurement. The tissue section was weighed again after drying for 72 h at 100 °C in an oven. The percentage of brain water content was calculated using the following formula: (wet weight − dry weight)/wet weight × 100%).

### Tissue Preparation

The 6-week-old rats were killed by an overdose of sodium pentobarbital and perfused via the left ventricle with ice-cold 0.1 M PBS. The brain was quickly removed from the skull, dissected into sections (cortex, hippocampus, and rest of the brain). Cortex and hippocampus sections were fixed with 10% formalin for 48 h at room temperature and rinsed with running tap water for 1 h. The tissue was dehydrated using a serial dilution of 70%, 80%, and 95% alcohol for 45 min each, three times with 100% alcohol for 1 h each, two times with xylene for 1 h each, and three times of paraffin for 1 h each. The tissue is then embedded in a paraffin block and sectioned into 6 µm thick slices on a microtome (RM2235, Leica, Germany). The ipsilateral basal ganglia section was taken out to be used for cerebral water content measurement. The rest of the brain was washed once with ice-cold PBS and cut into pieces on an ice bucket, mixed and divided for mitochondria, protein, and RNA extraction.

### H&E Stain

For H&E staining, sections were deparaffinized in xylene twice, each time for 10 min. The brain sections were then rehydrated twice in 100% ethanol for 5 min each, twice in 95% ethanol for 3 min each, once in 85% ethanol for 3 min, once in 75% ethanol for 3 min, and washed in distilled water for 2 min. The sections were stained in hematoxylin solution for 10 min, washed in running tap water for 1 min, incubated in 1% acid alcohol for 30 s, washed in running tap water for 1 min, incubated in 0.2% ammonia water for 30 s, washed in running water for 1 min, stained in eosin-phloxine solution for 3 min and dipped in water twice. Then the sections were dehydrated once in 80% ethanol for 20 s, twice in 95% ethanol for 3 min each, twice in 100% ethanol for 5 min each, three times in xylene for 5 min each, and mounted with a xylene-based mounting medium.

### Immunofluorescence

Sections were deparaffinized and placed in antigen retrieval buffer (brought to a boiling point in the microwave) and cooled off to room temperature. The slides were then washed with deionized water and 1X tris-saline buffer plus Tween 20. The slides were blocked with 5% bovine serum albumin (BSA) for 1 h at room temperature followed by three times of washing using washing buffer for 5 min each. The slides were then incubated overnight with primary FAM3A antibody (MA5-32880, ThermoFisher). The next day, the slides were washed 3 times using washing buffer and incubated in Alexa Fluor 594 secondary antibody (ab154207, Abcam) for 1 h at room temperature. The slides were washed 3 × with washing buffer and mounted with 4′,6-diamidino-2-phenylindole (DAPI) (P36935, Invitrogen) and a coverslip. The images of CA1 were taken in 6 random fields at ×400 magnification (*n* = 6 per group, 3 slices per animal) using as confocal microscope (TCS SP8 STED, Leica, Germany).

### Mitochondrial Isolation

Mitochondria were isolated from fresh brain tissues using a mitochondrial isolation kit (C3606 Biyuntian, China) according to manufacturer’s instructions. Specifically, the tissues were washed once with ice-cold PBS and cut into pieces on an ice bucket. The tissues were homogenized on ice with phenylmethylsulfonyl fluoride (PMSF) containing Solution A (1:10 ratio), and centrifuged at 11,000×*g* for 10 min at 4 °C. Crude mitochondrial pellet was resuspended in isolation buffer without ethylene glycol tetraacetic acid (EGTA) and the protein concentration was determined using a bicinochoninic acid (BCA) protein assay kit.

### Mitochondrial Function Assessment

ATP levels were measured according to manufacturer’s instructions (A095 Nanjing Jiancheng Bioengineering Research Institute, Nanjing China). Mitochondrial respiratory complex (I, II, III, and IV) activities were determined using kits from Nanjing Jiancheng (A089-1, A089-2, A089-3, and A089-4, Nanjing Jiancheng Bioengineering Research Institute, Nanjing, China), according to manufacturer’s instructions. The mitochondrial membrane potential was determined with JC-1 kit (HY-K0601, MedChem Express, Shanghai, China) via flow cytometry (NovoCyte, ACEA Biosciences). ROS production was measured using a kit from Nanjing Jiancheng Bioengineering Research Institute, China (E004-1-1) according to manufacturer’s instructions.

### ELISA

Brain tissues were homogenized with Tris–HCl buffer containing 1% pH 7.6 3-[(3-cholamidopropyl) dimethylammonio] Propanesulfonate and incubated at 4 °C for 3 h. The homogenate was then centrifuged at 22,000×*g* at 4 °C for 20 min. The supernatant was diluted with enzyme immunoassay buffer with 1% bovine serum albumin and 0.05% Tween-20 in PBS at 1:5 ratio. Protein concentration was measured using BCA protein assay kit. TNF-α and IFN-γ levels were measured using an ELISA kit (CSB-E11987r and CSB-E04579r, Wuhan Huamei, China), according to manufacturer’s instructions.

### Superoxide Dismutase (SOD) Activity

SOD activity was measured according to previously published protocol (Sun et al. 1988) with the T-SOD activity kit (Nanjing Jiancheng, A001-1-1). SOD activity was measured at 550 nm using Beckman DU 640 BV spectrophotometer.

### Real-Time RT-PCR

Total RNA extracted using TRIzol and synthesized into cDNA using a reverse transcription kit (Takara, Dalian, China) according to manufacturer’s guidelines. Real-time quantitative PCR was performed on cDNA with the Bio-Rad iQ5 Gradient Real-time PCR system (Bio-Rad Laboratories). The values obtained for FAM3A (F: 5′-GTCATACACCTTGTGAGGGACT-3′, R: 5′-TTTCAAGAAACTCTCCATCCC-3′) mRNA were normalized against the GAPDH (F: 5’-AAG GTG AAG GTC GGA GTC AA-3′, R: 5′-AAT GAA GGG GTC ATT GAT GG-3′) reference gene using the 2 − ΔΔCT method (Livak and Schmittgen, 2001). qPCR reactions were performed in triplicates with amplification efficiency of 90 = 95%. Specific parameters for the reaction are as follows: 95 °C for 3 min, 35 cycles of (94 °C for 30 s, 60 °C for 30 s, 72 °C for 30 s), and 72 °C for 10 min.

### Western Blotting

Protein lysates were extracted from brain tissues using boiling buffer. Protein concentration was measured using BCA protein assay kit. Forty micrograms of protein was resolved on 12% SDS-PAGE gel and transferred to polyvinylidene fluoride (PVDF) membranes. Membranes were blocked with 5% skim milk solution and incubated overnight at 4 °C with FAM3A (Thermo MA5-32880) or GAPDH (MA5-15738) antibodies diluted in TBST. The membranes were then washed and incubated with secondary antibody for 1 h at room temperature. Immunoreactivity was detected with Super Signal West Pico Chemiluminescent Substrate (Thermo Scientific, Rockford, IL, USA) and quantified with ImageJ (Scion Corporation).

### Statistics

Data are presented as mean ± SD. Unpaired *t* test was used to analyze differences observed between Ctrl-LV-Ctrl and HI-LV-FAM3A as well as HI-LV-FAM3A and HI-LV-Ctrl groups. Behavioral data were two-way repeated measures ANOVA using IBM SPSS Statistics 22. Data were considered significant when *p* value < 0.05 (*), < 0.01(**), < 0.005(***), < 0.001(****). All statistics were analyzed and graphed with the GraphPad Prism9 package.

## Results

### Hypoxia–Ischemia Inhibits FAM3A Expression and Contributes to Increased Mortality Rate

**Fig. 1:**
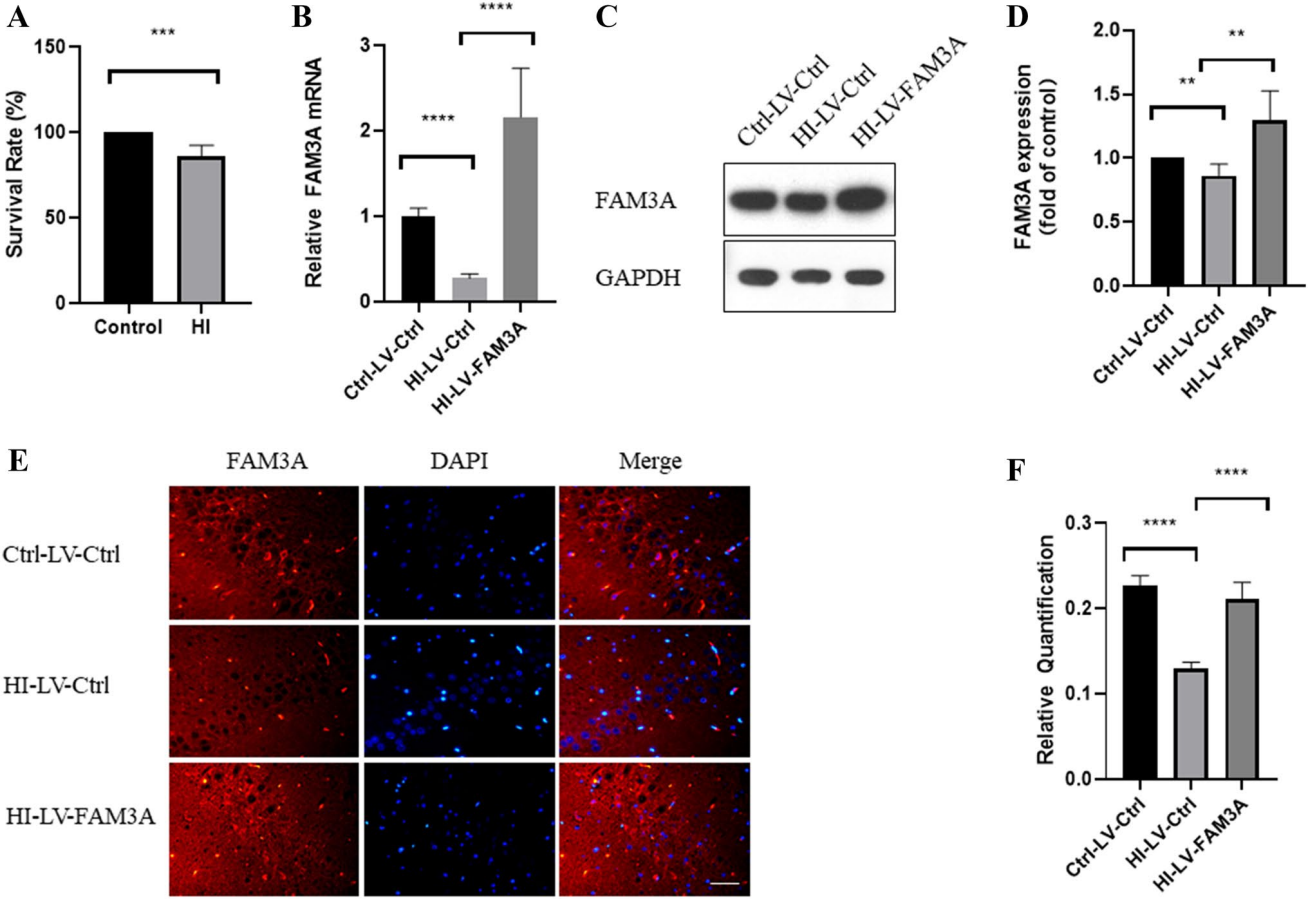
HI inhibits FAM3A expression and contributes to increased mortality rate. **A** survival rate of pups born in the control were significantly higher than that of the HI group. **B** mRNA level of FAM3A in brain tissues show decreased FAM3A expression, lentivirus transduction showed increased FAM3A expression. **C** and **D** image and quantification of FAM3A protein expression. **E** and **F** image and quantification of immunofluorescence of FAM3A in CA1 using confocal microscope. *n* = 6 animals from each group 6 fields were taken for 3 slices per animal, *p < 0.05, **p < 0.01, ***p < 0.005, ****p < 0.001

Through bilateral uterine artery ligation, which restricts blood flow to the fetuses, we were able to establish a HI rat model. As shown in Fig. 1A, all control pups showed 100% survival. And as expected, surgically-induced HI resulted in fewer surviving pups (86%), suggesting that gestational hypoxia–ischemia contributes to higher mortality rates. To examine the effect of HI on FAM3A expression levels, we established three different in vivo models through lentiviral transduction after the pups were born: non-HI with control lentivirus (Ctrl-LV-Ctrl), HI with control lentivirus (HI-LV-Ctrl), and HI with FAM3A lentivirus (HI-LV-FAM3A). Next, we extracted protein and mRNA from the animals’ brain tissues, and demonstrated that HI significantly reduces FAM3A expression through western blotting, RT-qPCR, and immunofluorescence assays (Fig. 1B–F). In addition, as shown by western blotting, qPCR, and immunofluorescence, the HI-LV-FAM3A group demonstrated significantly higher FAM3A expression levels (Fig. 1B–F).

### FAM3A Expression Contributes to Improved Neuronal Cell Morphology and Cognitive Function

**Fig. 2:**
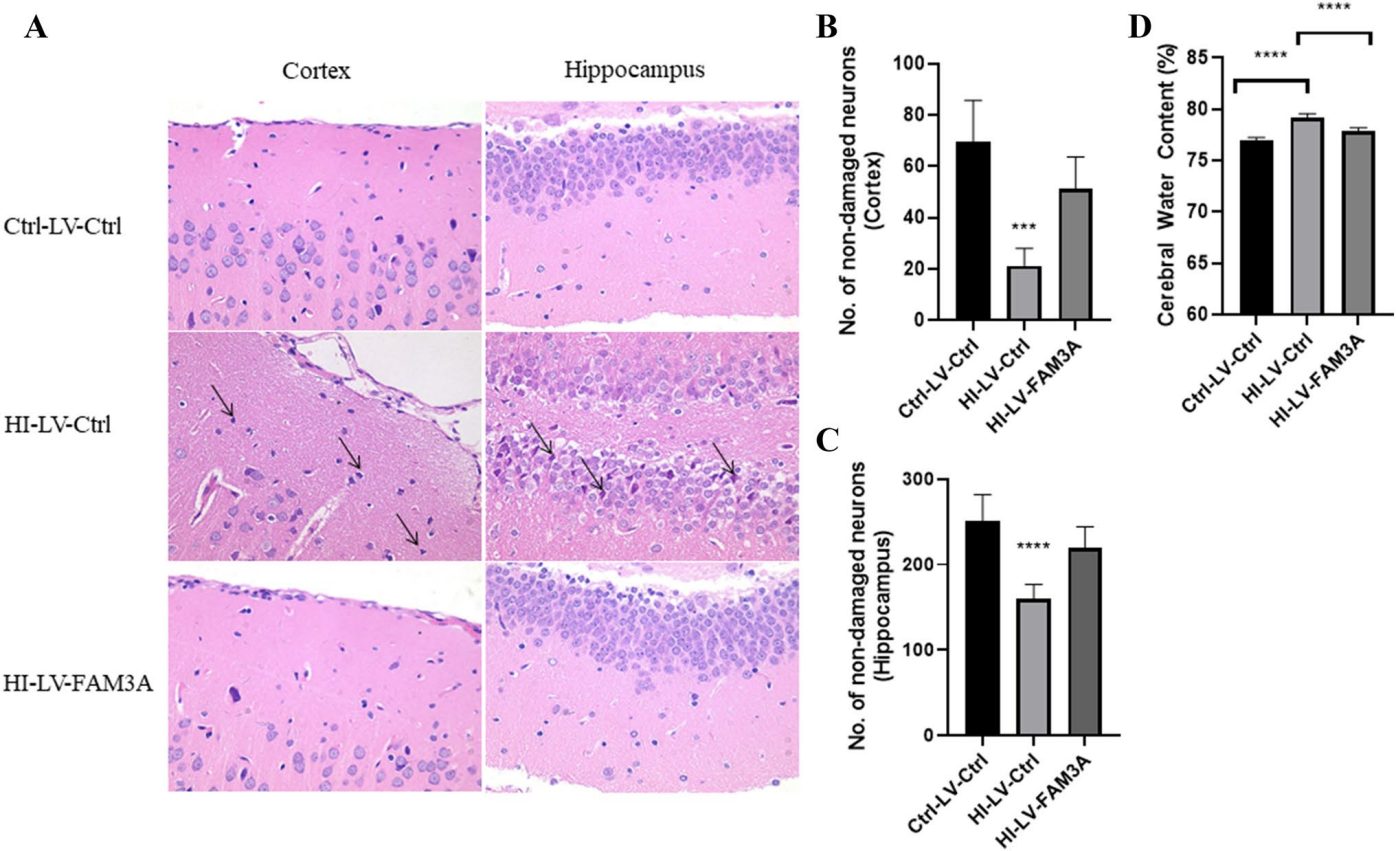
FAM3A expression contributes to improved neuronal cell morphology. **A** neuronal cell morphology in the cortex and hippocampus regions of the brain were examined through H&E staining. Neuronal cells in the HI group showed dark staining and small pyknotic nuclei, while control and FAM3A overexpressing groups demonstrated normal and round nuclei. Original magnification: ×400. **B** and **C** number of non-damaged neurons in the cortex and hippocampus. HI-LV-Ctrl group shows significantly less number of non-damaged cells compared to the Ctrl-LV-Ctrl and HI-LV-FAM3A groups. **D** % of cerebral water content. HI group demonstrated a 2.3% increase in cerebral water content, while FAM3A overexpression led to a 1.3% reduction. *n* = 6 animals from each group, *p < 0.05, **p < 0.01, ***p < 0.005, ****p < 0.001

First, we examined the potential effects of HI as well as FAM3A expression on neuronal cell morphology. Hematoxylin and eosin (H&E) staining was performed to examine neuronal cell damages in the cortex and the hippocampus. As shown in Fig. 2A, cells in the cortex and hippocampus of the control and FAM3A expressing rat brain tissues were neatly arranged with normal morphological structures as well as round nuclei, and clear nucleoli (*p* < 0.005). On the contrary, neuronal cells in the HI group were irregularly arranged with dark staining small pyknotic nuclei. Figure 2B and C show that the number of non-damaged cells is significantly less in the HI-LV-Ctrl group compared to the Ctrl-LV-Ctrl and HI-LV-FAM3A groups. Furthermore, brain water content, which measures cerebral edema and a major contributor to poor neurological conditions, was higher in the HI group compared to the control group (76.9% vs 79.2%) (*p* < 0.001) (Fig. 2D). Conversely, FAM3A overexpression was shown to be able to reduce brain water content (77.9) (*p* < 0.001) in the HI-induced rats. Both neuronal cell damage and increased cerebral water content serve to reflect some degree of brain injury.

**Fig. 3:**
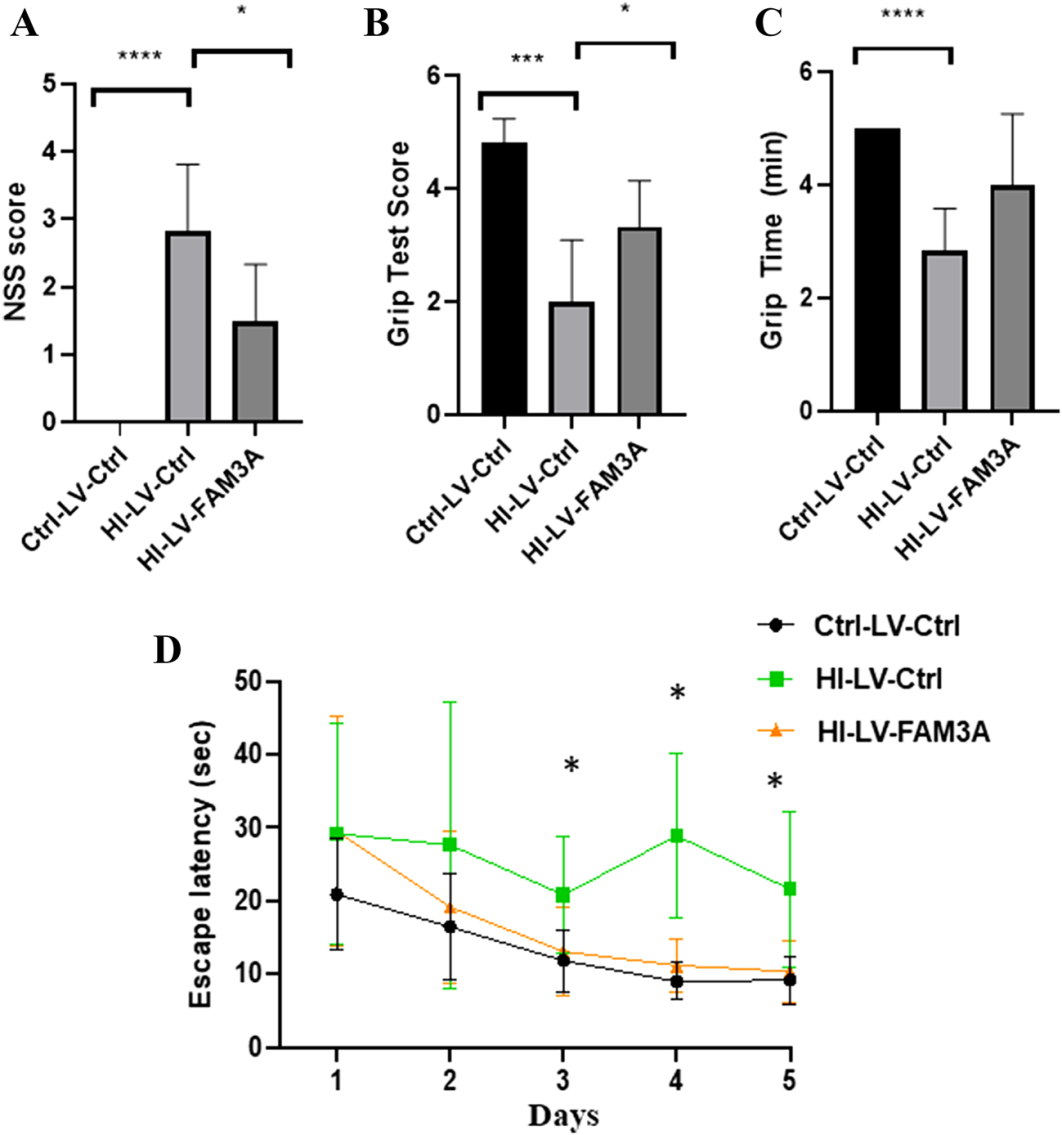
The effect of HI and FAM3A expression on memory, spatial learning, and motor function. **A** cognitive function assessments were assessed when the animals were 6.5 weeks-old. NSS evaluation was based on 10-point system, with high scores indicative of more severe neurological impairments. NSS values for Ctrl-LV-Ctrl, HI-LV-Ctrl, and HI-LV-FAM3A are 0, 2.83, and 1.5, respectively. **B** and **C** grip test was performed at 6.5 weeks, both scores and grip time were recorded. Both overall grip score and amount of time spent holding onto the wire were significantly less in the HI group compared to the control group. HI-LV-FAM3A rats performed significantly better than the HI-LV-Ctrl rats on grip score and grip time. **D** the escape latency period in the Morris water maze test showed that rats in the HI group required more time to escape platform compared to the control group, and that HI-LV-FAM3A group required less time compared to the HI-LV-Ctrl group. *n* = 6 animals from each group, *p < 0.05, **p < 0.01, ***p < 0.005, ****p < 0.001

**Fig. 4:**
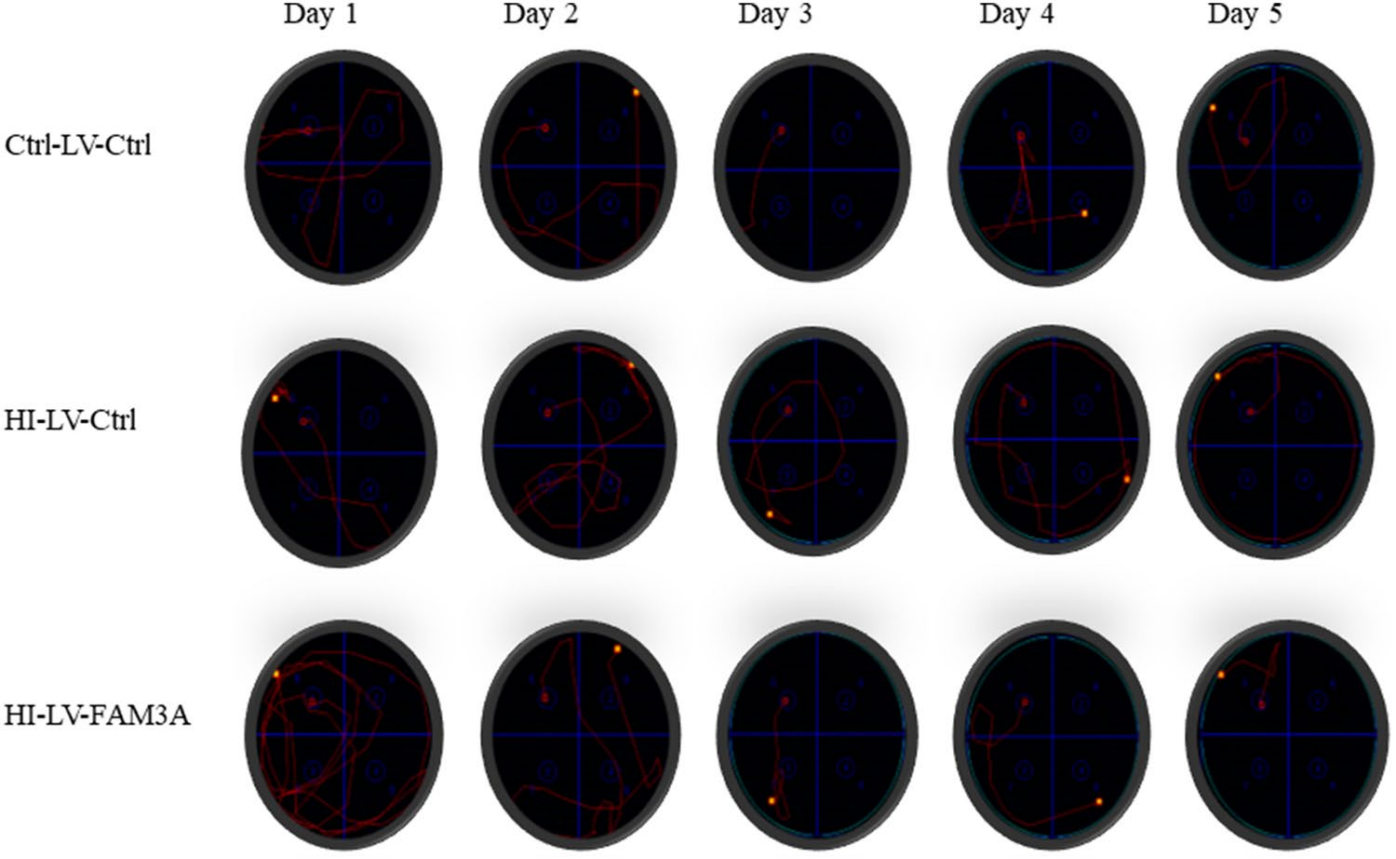
Swimming tracts for the Morris Water Maze test. Representative swimming tracks in the Morris water maze for days 1–5

Next, we investigated the impact of HI-induced brain injury on specific cognitive and motor functions. We first performed a neurological severity score (NSS) assessment. Figure 3A depicts the mean NSS values for all three groups. Compared to the control group (0), the mean NSS value for the HI rat was significantly higher (2.8, *p* < 0.001). While overexpression of FAM3A did not reduce NSS values to the level of the control group, but compared to the HI-LV-Ctrl group, FAM3A expression was able to improve overall NSS (1.5, *p* < 0.05). Overall, these results indicate that HI can elicit cognitive impairments as reflected by poor NSS, increased cerebral water content, and damaged neuronal cells, and that increased FAM3A expression may have rescuing effects. Next, the hanging wire grip test was performed to assess motor functional deficits caused by HI. The grip test score is based on how firmly and whether both forelimbs and hindlimbs were used to hold onto the wire. As shown in Fig. 3B, C, both overall grip score and latency to fall period for the Ctrl-LV-Ctrl were superior than the HI-LV-Ctrl group. Furthermore, animals in the group with FAM3A overexpression demonstrated stronger forelimb and hindlimb strength. In addition, the Morris water maze test, which measures reference memory and hippocampus-dependent spatial navigation, was performed to assess potential changes in spatial learning and memory. In the five advancing training days, while the escape latency period for the Ctrl-LV-Ctrl and HI-LV-FAM3A groups gradually decreased, rats in the HI-LV-Ctrl group did not show shorter path lengths and escape latencies (Fig. 3D). Specifically, by day 3 (*p* < 0.05) and day 5 (*p* < 0.05), the escape latency period for the Ctrl-LV-Ctrl and HI-LV-FAM3A groups were both significantly shorter compared to the HI-LV-Ctrl group. Swimming tracks were recorded for each group on each day, a representative figure is shown in Fig. 4. This result indicates that HI-induced brain injury negatively impacts memory and spatial learning. These results suggest that HI inhibits spatial learning and memory as well as motor function, while overexpressing FAM3A demonstrated improved outcomes.

### HI Inhibits While FAM3A Enhances Mitochondrial Function

**Fig. 5:**
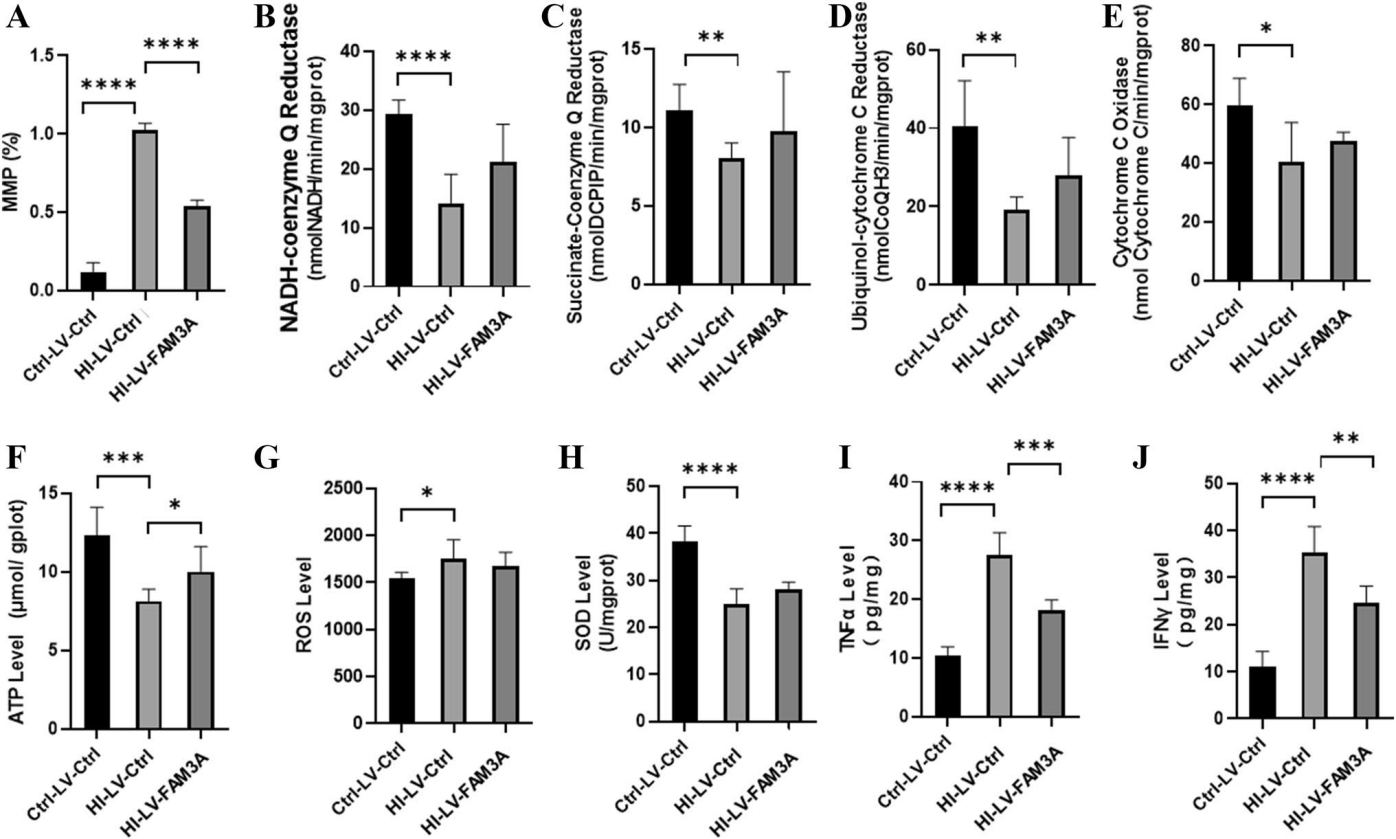
HI inhibits while FAM3A enhances mitochondrial function. **A** membrane potential was assessed using JC-1 dye. The percentage reflects the ratio of JC-1 aggregates and JC-1 molecules, the higher the percentage is indicative of lower membrane potential. The HI-LV-Ctrl group exhibited lower membrane potential compared to Ctrl-LV-Ctrl and HI-LV-FAM3A groups. **B**–**E** activities of mitochondrial complexes I-IV (NADH-coenzyme Q reductase, succinate-coenzyme Q reductase, ubiquinol-cytochrome C reductase, and cytochrome C oxidase) were reduced in HI rats, and FAM3A expression did not significantly promote their activity levels. **F** ATP level in the HI-LV-Ctrl group was significantly lower than that of the Ctrl-LV-Ctrl and HI-LV-FAM3A group. **G** and **H** ROS levels and SOD activity were higher in the HI-LV-Ctrl group compared to the Ctrl-LV-Ctrl group, but not significantly different from HI-LV-FAM3A group. **I** and **J** ELISA results show heightened TNF-α and IFN-γ levels in the HI-LV-Ctrl group compared to the Ctrl-LV-Ctrl as well as the HI-LV-FAM3A group. *n* = 6 animals from each group, *p < 0.05, **p < 0.01, ***p < 0.005, ****p < 0.001

Accumulating evidence suggests that maintaining functional mitochondria is critical for neuronal cell survival (Liu et al. 2018; Huang et al. 2016). Given the role of FAM3A as a mitochondrial protein, we further examined potential changes in mitochondrial function as a result of HI. Results show that rats in the HI group demonstrated a significant reduction in mitochondria membrane potential (MMP) as compared to the control group (*p* < 0.001), and FAM3A overexpression was able to promote MMP levels (*p* < 0.001) (Fig. 5A). We also examined potential changes in the transmembrane protein complex I (NADH ubiquinone oxidoreductase), complex II (succinate dehydrogenase, complex III (ubiquinol cytochrome c oxidoreductase, and complex IV (cytochrome c oxidase), which are important factors in the mitochondrial electron transport chain (ETC), and are necessary for generating the electrochemical gradient for ATP biosynthesis. As shown in Fig. 5B–E, levels of all four complexes were reduced in the HI rats. However, overexpression of FAM3A did not seem to shown any rescuing effect on their levels. Changes in MMP and transmembrane protein complexes further prompted us to investigate the effect of HI and FAM3A expression on ATP and ROS production. As shown in Fig. 5F, rats subjected to HI demonstrated significantly reduced ATP production compared to the control group rats (*p* < 0.005), and overexpression of FAM3A was shown to promote ATP levels in the HI rats (*p* < 0.05). Interestingly, while ROS level increased as a result of HI (*p* < 0.05), overexpression of FAM3A did not show a rescuing effect. This indicates that the rescuing effect of FAM3A may not be through limiting ROS production (Fig. 5G). In accordance with this result, we also find that FAM3A expression was unable to effectively enhance innate antioxidant defense system. In particular, with increased ROS levels, it is expected that the activity of antioxidant scavengers such as superoxide dismutases (SODs) would increase. However, contrary to our expectation, SOD activity was significantly decreased in the HI group (*p* < 0.001), and FAM3A expression did not seem to rescue its activity level (Fig. 5H). These results suggest that while HI may inhibit SOD activity, the effect of overexpressing FAM3A on limiting ROS generation through enhancing SOD activity may be limited. Previous studies have reported increases in TNF-α and IFN-γ levels after permanent cerebral brain ischemia (Yilmaz et al. 2006; Doll et al. 2015). In C. *rodentium* and E. *coli*, expression of TNF-α and IFN-γ were shown to negatively regulate mitochondrial function (Maiti et al. 2015). In agreement with these reports, our results showed that rats in the HI group demonstrated significant increases in TNF-α (*p* < 0.001) and IFN-γ (*p* < 0.001) levels (Fig. 5I, J). Interestingly, overexpression of FAM3A was able to significantly repress levels of both in the HI rats. These data suggest that the rescuing effect of FAM3A may be reflected through its function in repressing TNF-α and IFN-γ levels.

## Discussion

In this study, we provided evidence that HI-induced brain injury is correlated with inhibited FAM3A expression, and that overexpression of FAM3A was able to mitigate HI-induced neuronal cell damage, cognitive, and motor dysfunction in vivo. Our results are also in accordance with previous study demonstrating the protective role of the Snhg8/miR-384/Hoxa13/FAM3A axis in cerebral ischemia-induced neuronal apoptosis (Liu et al. 2019). As shown in this study, HI was detrimental to survival rate. Moreover, surviving progenies demonstrated both neuronal cell damage as well as thwarted motor and cognitive functions. Notably, as a result of HI, neuronal cells in the cortex and hippocampus regions were shown to be irregularly arranged along with small pyknotic nuclei, which serve as a reflection of irreversible chromatin condensation in the nucleus as well as cellular apoptosis. In addition, our results indicated that HI promoted brain edema, a contributor to poor neurological outcomes, as reflected by a 2.3% increase in cerebral water content. While the percentage increase is modest, but as reported in other global ischemia studies, a 1% increase in cerebral water content can lead to profound changes in intracranial pressure, which is a direct reflection of restricted blood supply and brain injury. Moreover, through a series of behavioral tests, we showed that cognitive and motor functions were significantly altered in HI rats, as illustrated by higher NSS scores, escape latency periods, and reduced limb strength. Alternatively, HI rats administered with FAM3A overexpression demonstrated lower NSS scores and improved performance on the hanging wire grip and morris water maze tests, accompanied by reduced neuronal cell damage and cerebral water content. While the improvements were significant compared to the HI-LV-Ctrl rats, there was still some difference between the Ctrl-LV-Ctrl and HI-LV-FAM3A group in all the behavioral tests, although not statistically significant. This suggests that while FAM3A may be able to improve HI-induced brain impairment, future studies should evaluate whether time, dose (multiple injection), and method of FAM3A administration can affect the degree of cognitive improvement in HI rats.

Accumulating evidence has suggested that maintaining normal mitochondrial function is vital for neuron survival (Wang et al. 2021). Mitochondria are multifunctional organelles and critically important for cell survival as they not only play a key role in generating ATP through oxidative phosphorylation, but are also responsible for maintaining membrane potential, producing ROS, and mediating intracellular calcium buffer. The central nervous system, which accounts for 2% of body weight, has an extraordinarily high metabolic demand and consumes 20% of total inspired oxygen (Kann and Kovacs 2007; Silver and Erecinska 1988). Since neurons cannot store excess energy, the large amount of ATP needed for executing neurotransmission and survival depend on continuous oxygen supply and functional mitochondria (Ames 2000; Erecinska et al. 2004; Mayevsky and Chance 1975). Mitochondrial malfunction can occur within minutes of reduced blood and oxygen supply in the brain (Liu et al. 2018). Of note, immediately after HI-induced impaired cerebral blood flow, ATP is depleted, and a cascade of events including membrane ion pumps failure due to lack of energy, influx of sodium, efflux of potassium, membrane depolarization and increased release of voltage-dependent mitochondrial Ca2+ , will lead to higher free radical production and activated cell death signals and inflammatory mediators (Hofmeijer and van Putten, 2012; Dharmasaroja 2016). In fact, research has shown that post traumatic brain injury, both structural and functional damage of the mitochondria can contribute to impaired cognitive function and neuronal cell death (Fischer et al. 2016; Lifshitz et al. 2004; Singh et al. 2006; Cheng et al. 2012; Gajavelli et al. 2015). Therefore, targeting mitochondria is one of the promising neuroprotective strategies for HI-induced brain injury treatment (Huang et al. 2016).

A key feature of the mitochondria is to maintain the ΔΨm at around − 180 mV. The mitochondrial membrane potential, which provides the driving force for electron transport, along with the proton gradient (ΔpH), forms the transmembrane potential of hydrogen ions necessary for making ATP (Zorova et al. 2018). ΔΨm levels fluctuate within limited range during normal physiological activities; however, prolonged rise or drop in ΔΨm can lead to the release of mitochondrial apoptosis initiation factors (AIFs) (Zorova et al. 2018). Our data show that HI significantly increased MMP levels, and that the abnormal change in MMP is coupled with increased ROS generation and reduced ATP production. Interestingly, overexpression of FAM3A was able to promote ATP production and reduce ΔΨm levels back to normal; however, HI-induced ROS escalation could not be mitigated by FAM3A expression. Under normal circumstances, antioxidant systems such as superoxide dismutase reacting with superoxide radicals followed by breaking down by glutathione peroxidase and catalase (Ham and Raju 2017; Winterbourn 2013), are adequate for removing ROS generated through cellular metabolism. However, HI insult can dramatically escalate ROS production and overwhelm mitochondria’s antioxidant capacity. As our data show, FAM3A was unable to promote SOD activity nor the activity of mitochondrial respiratory complexes I-IV, which may be the reason for sustained ROS production. These data suggest that the rescuing effect of FAM3A on mitochondrial function may be targeted towards regulation of mitochondrial membrane potential and ATP production.

To explore possible factors mediating the effect of FAM3A on mitochondrial function, we investigated potential changes in the brain’s inflammatory response. The intricate relationship between inflammatory response and mitochondrial dysfunction has been evidenced in multiple neurological diseases (van Horssen et al. 2019). In particular, TNF-α has been shown to correlate with poor clinical outcomes after ischemic stroke. In HT-22 cells, treatment with TNF-α was able to elicit mitochondrial dysfunction and neurotoxic effects (Doll et al. 2015), and experimental studies have suggested that TNF can trigger complex I, II, IV, as well as ATP synthase activity (Moe et al. 2004; van Horssen et al. 2019). Moreover, IFN-γ has also be demonstrated to inhibit mitochondrial function by promoting ROS producing and decreasing complex I and IV activity through the nitric oxide pathway (Maiti et al. 2015). As our results show, escalated levels of TNF-α and IFN-γ post HI were significantly reduced by overexpressing FAM3A, which suggests that FAM3A may play a role in regulating mitochondrial function through inhibition of inflammatory cytokines.

Previous research has predominantly focused on the role of FAM3A in insulin resistance, gluconeogenesis, lipogenesis, hypertension, chondrocyte apoptosis, and cardiovascular hypertrophy (Chen et al. 2020; Xiang et al. 2020; Yan et al. 2019; Yang et al. 2020). A study led by Xu et al. explored FAM3A suppression under hypoxic conditions in relation to endothelial angiogenesis and ischemic vascular disease, and Liu et al. demonstrated the network effects of Snhg8/miR-384/Hoxa13/FAM3A on neuronal apoptosis using ischemic mice model (Xu et al. 2019; Liu et al. 2019). Our study utilizes ischemic rat model to examine the mediating effects of FAM3A expression on brain impairment both from morphological and cognitive points of view. Moreover, rather than direct induction of HI on the neonates, we employed uterine artery bilateral ligation on the pregnant rats to focus on the effect of maternal stress on the offspring’s FAM3A levels and whether overexpression of FAM3A postnatally could ameliorate brain impairments in the neonatal rats. And as a mitochondrial protein, we also demonstrated that FAM3A can enhance cerebral mitochondrial function in vivo, potentially to the benefit of FAM3A-mediated brain recovery in terms of both neuronal cell morphology and cognitive function. Overall, results from our study reflect the protective effects of FAM3A on neonatal rat brain impairment post HI induction during gestation. While we provide preliminary evidence indicating the potential for FAM3A to serve as a new clinical target for HI recovery, further confirmation in additional in vitro and in vivo models is necessary. Future studies may look into the difference in timing as well as technique in safe induction of FAM3A overexpression in neonates.

## Conclusion

Prenatal stage is a critical period for brain development, any disruptions can lead to irreversible damage to the neuronal network, compromise cognitive functions, and predispose patients to neurodegenerative diseases later in life. A growing body of literature from both clinical and experimental brain injury research has shown that structural and functional damage of mitochondria is an early event after HI brain injury, which contributes to cell death and poor cognitive outcome. We demonstrate that FAM3A is capable of exerting mitigating effects for neuronal cell damage, as well as cognitive and motor impairments post HI. And more important, we provide evidence that FAM3A may regulate mitochondrial function, specifically, changes in membrane potential and ATP production, through pro-inflammatory cytokines. Future studies involving the mechanism TNF-α and IFN-γ regulation through FAM3A is warranted at the molecular level.

## Limitations of this Study

Although our study provides in vivo evidence, it would be strengthened by showing the effect of FAM3A on neuronal cell survival through regulation of mitochondrial activity and of pro-inflammatory cytokines TNF-α and IFN-γ at the cellular level. The study was also limited in that the number of animals used in each group was small, which warrants future studies to examine the protective roles of FAM3A in other animal models and in larger numbers. In addition, considering that different regions of the brain have differential susceptibility to ischemia, our results may be limited in that the hippocampus and cortex regions were used for H& E and immunohistochemical staining, while the rest of the brain were used for the other experiments (PCR, ELISA, Western Blotting, etc.). Moreover, a Ctrl-LV-FAM3A group would have provided more concrete evidence for the findings presented in this study. Lastly, tap water contains iron, sulfur, and chlorine, which can alter the pH of the water and may affect the bluing agent in the H&E stain.

## Data Availability

The datasets used and analyzed during the course of this study are available from the corresponding author upon reasonable request.
